# The role of playing position in soccer injury characteristics: evidence from sub-elite athletes

**DOI:** 10.3389/fspor.2025.1542300

**Published:** 2025-02-17

**Authors:** Makete Thomas Thema, Suzanne Jacobs, Linda van den Berg, Anita Strauss, Mzwandile Prescott Mahlangu

**Affiliations:** Department of Sport, Rehabilitation and Dental Sciences, Faculty of Science, Tshwane University of Technology, Pretoria, South Africa

**Keywords:** soccer injuries, playing position, sub-elite athletes, injury prevention, South Africa

## Abstract

This study examines the association between playing position and injury characteristics among sub-elite male soccer players in South Africa. Using a cross-sectional survey, 223 players from four universities were assessed for injury prevalence, type, mechanism, and severity during the 2023 soccer season. Midfielders experienced the highest injury frequency (43.6%), followed by defenders (30.0%), forwards (17.9%), and goalkeepers (8.6%). Lower limb injuries were predominant across all positions (89.6%), with defenders (94.6%) and midfielders (95.1%) at greatest risk, while goalkeepers sustained a significant proportion of upper limb injuries (44.4%) due to their specialized role. Soft tissue injuries were most common among midfielders (78.0%) and defenders (67.6%), whereas goalkeepers reported higher rates of bone-related injuries (66.7%). Defensive actions, such as tackling, accounted for most injuries among defenders (56.8%), while aerial play contributed substantially to goalkeeper injuries. No statistically significant differences in injury severity were found across positions. These findings highlight the influence of playing position on injury characteristics and underscore the need for position-specific injury prevention strategies tailored to sub-elite soccer players.

## 1 Introduction

Soccer, widely recognized as the most popular sport globally, boasts over 240 million participants across more than 200 nations (1, 2). Despite its appeal and numerous benefits, soccer presents significant injury risks, particularly among sub-elite players who lack the resources and protective measures available to their elite counterparts. Elite players typically benefit from advanced medical facilities, professional physiotherapy, individualized strength and conditioning programs, access to sports science technology (e.g., GPS tracking and load monitoring), and comprehensive injury prevention strategies, which are often unavailable to sub-elite athletes (3, 4). Access to sports physiotherapy ensures appropriate rehabilitation for injuries, while conditioning programs tailored to player-specific needs help improve strength, flexibility, and endurance. Advanced tools such as GPS monitoring aid in managing player workload, thereby minimizing overuse injuries (3, 5). Furthermore, structured warm-up programs such as the FIFA 11+ have been shown to reduce injury risk by enhancing neuromuscular control (2, 4).

Research into injury epidemiology in soccer has primarily focused on elite and professional players, often neglecting sub-elite athletes, who represent a competitive tier between amateur and professional levels (6, 7). Sub-elite players frequently participate in intense competition and training sessions without access to the same medical and conditioning resources, leaving them more vulnerable to injuries.

The occurrence of injuries in soccer is influenced by several factors, including the physical demands of the sport, environmental conditions, and intrinsic player characteristics. Characteristics associated with player position have been linked with injury susceptibility, as the role dictates the intensity, frequency, and type of physical activities performed during matches. Positional demands often lead to differing injury risks among players, with defenders, midfielders, and forwards exposed to unique challenges due to their roles in tackling, ball control, or offensive strategies (8, 9). Sarmento et al. (10), in their systematic review, found that positional roles in soccer significantly influence physical, physiological, and technical demands, with midfielders and external defenders covering greater distances at high speeds and performing more sprints, while central defenders and midfielders executed more passes, emphasizing the importance of position-specific training practices.

Seminal research has highlighted positional variations in injury risk, with Ekstrand et al. (3) showing that midfielders experience the highest injury rates due to their extensive field coverage and physical demands, while Junge & Dvorak (4) identified positional-specific mechanisms contributing to injury susceptibility. Overall, characteristics associated with the physicality of their role predispose defenders to a higher injury risk, particularly due to the demands of tackling and other high-impact actions (11, 12).

Theoretical perspectives suggest that positional demands in soccer directly influence injury mechanisms. Midfielders experience a high prevalence of overuse injuries due to their extensive field coverage and frequent high-intensity actions. For example, Ekstrand et al. (3) reported that midfielders accounted for 40% of overuse injuries in elite European soccer. Similarly, Della Villa et al. (13) and Rahnama et al. (14) highlighted that the physical and cognitive demands placed on midfielders significantly contribute to repetitive strain injuries, such as hamstring and groin strains, which are among the most common (13, 14).

Goalkeepers, in contrast, are exposed to acute injury risks due to explosive actions like diving, jumping, and reaction-based movements, which often lead to upper limb injuries (4, 11). Positional demands also vary in their cognitive requirements, with midfielders managing complex decision-making under sustained physical exertion, further compounding their injury susceptibility (15).

In South Africa, the lack of comprehensive research on soccer injuries at the sub-elite level further exacerbates the challenge of implementing effective injury prevention strategies. While South African universities actively participate in soccer leagues with growing player enrollment, the use of scientific methods to address injuries remains limited (16). Although focused on professional players, Calligeris et al. (16) offers a useful baseline for comparing injury patterns, emphasizing the unique challenges sub-elite players face due to limited access to injury prevention resources. This gap highlights the need to analyze injury patterns specific to this demographic to improve player safety and performance.

This study aims to explore the characteristics of soccer injuries concerning playing positions among sub-elite male athletes in South Africa. Existing injury prevention programs, such as the FIFA 11+, could be adapted and tailored to suit the specific needs of sub-elite players in this context.

By examining the prevalence, mechanisms, and types of injuries across different positions, the findings will provide valuable insights for developing targeted injury prevention programs tailored to the specific demands of sub-elite soccer players.

## 2 Materials and methods

### 2.1 Study design

This study utilized a cross-sectional survey design to examine the prevalence, mechanisms, and types of injuries experienced by sub-elite male soccer players. The design was selected for its ability to capture a snapshot of injury patterns and their associations with playing positions during a specific soccer season (17).

### 2.2 Study population and sampling

The study was conducted during the 2023 competitive season, with data collected between March and October from 223 male soccer players representing four South African universities: North-West University (Potchefstroom, Mahikeng), Tshwane University of Technology, Nelson Mandela University, and the University of Limpopo. The distribution of participants by playing position reflects the natural composition of soccer teams, where goalkeepers are fewer compared to outfield players. While this limits statistical power for certain subgroups, the overall sample remains representative of sub-elite soccer teams in South Africa. Players were purposively sampled, as they met specific inclusion criteria relevant to the study. The participants were registered students, aged 18 years or older, actively playing in university soccer teams under the jurisdiction of University Sport South Africa (USSA). Players who did not meet these criteria or declined to provide informed consent were excluded from the study.

### 2.3 Data collection instruments

Data were collected using a self-administered online questionnaire hosted on the Survey Monkey platform. The questionnaire, adapted from validated tools by Hawkins and Fuller (18) and Twizere (19), was divided into two sections. The first section collected demographic and player-specific information, such as age, playing position, dominant leg, and years of experience. The second section focused on injury details, including the number, type, and severity of injuries sustained, as well as their mechanisms and recurrence. Injuries were recorded separately for training sessions and matches to identify contextual variations. Injuries were categorized into time-loss injuries, defined as any physical complaint resulting in the inability to participate in soccer activities for at least 24 h, and non-time-loss injuries, which did not impede participation (20). Injury severity was categorized based on the duration of time lost from participation: minimal (1–3 days), mild (4–7 days), moderate (8–28 days), and severe (>28 days), as per the definitions established by Fuller et al. (20).

### 2.4 Data collection procedure

Participants were provided with a link to the questionnaire, accompanied by an information sheet explaining the study's purpose, procedures, and confidentiality measures. Only players who sustained injuries during the season were required to complete the injury-specific section. To minimize recall bias, players with multiple injuries were instructed to report on their most recent injury (21, 22). Injuries unrelated to soccer activities were excluded.

### 2.5 Ethical considerations

Ethical approval was obtained from the Tshwane University of Technology Research Ethics Committee (REC/2023/01/003). Participants provided informed consent via an online consent form embedded in the questionnaire. Anonymity was ensured through the assignment of numerical codes, and data access was restricted to the research team. Participants were informed of their right to withdraw at any time without penalty. To reduce the likelihood of the study findings influencing coaches' decisions about player participation, only team-level results (excluding individual data) were shared with the coaching staff.

### 2.6 Data analysis

Data were analyzed using IBM SPSS Statistics for Windows, Version 28.0 (IBM Corp., Armonk, NY, USA). Descriptive statistics summarized injury patterns and player demographics, while Pearson's chi-squared tests assessed associations between playing position and other variables, including injury type, injury location, mechanism of injury, and injury severity. Effect sizes (Cramér's V) were calculated for significant chi-squared results to quantify the strength of associations. Assumptions for the test, including a minimum expected cell frequency of 5, were verified and satisfied for all analyses. Statistical significance was set at *p* < 0.05 to identify meaningful associations between variables while maintaining a balance between Type I and Type II error risks. Given the exploratory nature of the study, no formal corrections for multiple comparisons were applied to avoid inflating the likelihood of false negatives. Injuries were expressed as time-loss injuries, defined as any injury resulting in the inability to participate in training sessions or matches for a specified duration. Subgroup analyses for goalkeepers and forwards were primarily descriptive due to smaller sample sizes, and the results should be interpreted cautiously in light of reduced statistical power.

### 2.7 Reliability and validity

The questionnaire demonstrated strong reliability and validity, as evidenced by Cronbach's alpha values exceeding 0.80 for internal consistency. Content validity was ensured through pre-testing with a pilot sample of sub-elite players. The pilot sample consisted of 15 sub-elite male soccer players, who provided feedback on the clarity, relevance, and ease of use of the questionnaire. The questionnaire was further validated by referencing established tools and literature in soccer injury research (18, 19).

## 3 Results

### 3.1 Player demographics

The study included 223 male sub-elite soccer players from four South African universities (Table 1). The players had an average age of 21.36 years, with the majority being right-leg dominant (68.2%). The positional distribution revealed that midfielders constituted the largest group (41.7%), followed by defenders (32.3%), forwards (17%), and goalkeepers (9%).

**Table 1:** Demographic data of participants.

Variable	Category	*n*	%
Age	Mean	21.36	SD = 2.55
Position	Defender	72	32.3
	Forward	38	17.0
	Goalkeeper	20	9.0
	Midfielder	93	41.7
	Total	223	100.0
Dominant leg	Both	26	11.7
	Left	45	20.2
	Right	152	68.2
	Total	223	100.0

### 3.2 Distribution of injuries by playing position

According to Table 2, midfielders experienced the highest proportion of injuries, accounting for 43.6% of all reported cases. A substantial portion of these injuries occurred during matches (40.2%), while a slightly higher percentage (49.1%) occurred during training. Defenders represented the second largest group, contributing to 30.0% of injuries, with 33.3% of these injuries sustained in matches and 24.5% in training. Forwards experienced 17.9% of the injuries, and goalkeepers accounted for the lowest percentage at 8.6%. These findings highlight the variations in injury rates across different playing positions.

**Table 2:** Distribution of injuries by playing position.

Position	Match		Training		Total	
	*n*	%	*n*	%	*n*	*%*
Defenders	29	33.3	13	24.5	42	30.0
Forwards	17	19.5	8	15.1	25	17.9
Goalkeepers	6	6.9	6	11.3	12	8.6
Midfielders	35	40.2	26	49.1	61	43.6
Total	**87**	**100**.**0**	**53**	**100**.**0**	**140**	**100**.**0**

### 3.3 Association between playing position and injury characteristics

The analysis in Table 3 revealed significant associations between playing position and injury characteristics.

**Table 3:** Association of playing position with injury characteristics.

Variable	Goalkeeper	Defender	Midfielder	Forward	Total	*X* ^2^	*p*-value
Body part							
Head to neck	0	0	0	1 (5.3%)	1 (0.9%)	–	–
Upper limb	4 (44.4%)	2 (5.4%)	2 (4.9%)	2 (10.5%)	10 (9.4%)	–	–
Lower limb	5 (55.6%)	35 (94.6%)	39 (95.1%)	16 (84.2)	95 (89.6%)	–	–
Total	**9**	**37**	**41**	**19**	**106**	**19.303**	* **0.004**
Type							
Soft Tissue	1 (11.1%)	25 (67.6%)	32 (78.0%)	9 (47.4%)	67 (63.2%)	–	–
Bone-related	6 (66.7%	8 (21.6%)	8 (12.2%)	9 (47.4%)	28 (63.2%)	–	–
Head	0	0	3 (7.3%)	0	3 (2.8%)	–	–
Pelvic and Pubic	2 (22.2%)	4 (10.8%	1 (2.4%)	1 (5.3%)	8 (7.5%)	–	–
Total	**9**	**37**	**41**	**19**	**106**	**27.692**	* **0.001**
Mechanism							
Physical actions	4 (44.4%)	12 (32.4%)	11 (26.8%)	3 (15.8%)	30 (28.3%)	–	–
Aerial Play	2 (22.2%)	1 (2.7%)	5 (12.2%)	1 (5.3%)	9 (8.5%)	–	–
Defensive actions	0	21 (56.8%)	16 (39.0%)	12 (63.2%)	49 (46.2%)	–	–
Ball vcontrol/movement	3 (33.3%)	0	5 (12.2%)	2 (10.5%)	10 (9.4%)	–	–
Physical demands	0 (0.0%)	3 (8.1%)	4 (9.8%)	1 (5.3%)	8 (7.5%)	–	–
Total	**9**	**37**	**41**	**19**	**106**	**23.561**	* **0.023**
Severity							
Minimal	1 (11.1%)	6 (16.2%)	2 (4.9%)	3 (15.8%)	12 (11.3%)	–	–
Mild	4 (44.4%)	8 (21.6%)	15 (36.6%)	5 (26.3%)	32 (30.2%)	–	–
Moderate	3 (33.3%)	8 (21.6%)	11 (26.8%)	6 (31.6%)	28 (26.4%)	–	–
Severe	1 (11.1%)	15 (40.5%)	13 (31.7%)	5 (26.3%)	34 (32.1%)	–	–
Total	**9**	**37**	**41**	**19**	**106**	**7.715**	**0.563**

*Note.* Values in brackets represent the percentage of the total within each playing position (values below 1% not shown).

* *p* < 0.05.

A significant association was found between playing position and injury location, *X*^2^ (3, *n* = 223) = 19.303, *p* = 0.004, Cramér's *V* = 0.38, indicating a moderate association. Lower limb injuries were most prevalent across all positions, especially for defenders (94.6%) and midfielders (95.1%), while goalkeepers showed a higher proportion of upper limb injuries (44.4%).

A strong association between playing position and injury type was observed, *X*^2^ (3, *n* = 223) = 27.692, *p* < 0.001, Cramér's *V* = 0.46. Soft tissue injuries predominated among midfielders (78.0%) and defenders (67.6%), while goalkeepers experienced more bone-related injuries (66.7%).

A significant moderate association was found between playing position and injury mechanism, *X^2^* (4, *n* = 223) = 23.561, *p* = 0.023, Cramér's *V* = 0.32. Defensive actions accounted for most injuries among defenders (56.8%), whereas aerial play was a prominent mechanism for goalkeepers (22.2%).

No significant association was found between playing position and injury severity, *X*^2^ (3, *n* = 223) = 7.715, *p* = 0.563, Cramér's *V* = 0.18. However, defenders reported a slightly higher proportion of severe injuries (40.5%).

## 4 Discussion

The findings of this study highlight the significant influence of playing position on injury frequency, location, type, mechanism, and severity among sub-elite soccer players. These results build upon and reinforce previous research while offering new and valuable insights.

### 4.1 Injury frequency by playing position

The injury distribution in our study aligns with prior research in sports medicine and injury epidemiology. Midfielders, frequently involved in offensive and defensive plays, experience the highest injury rates. Ekstrand et al. (3) reported midfielders accounted for 40% of injuries in elite European football, closely matching our finding of 43.6%. Similarly, Waldén et al. (23) observed defenders constituted 30% of injuries, consistent with our 30.0%. In contrast, forwards and goalkeepers have lower injury rates, as noted by Hägglund, Waldén, and Ekstrand (5), who reported 18% for forwards and 10% for goalkeepers, comparable to our findings of 17.9% and 8.6%, respectively. Some studies report differing injury distributions across positions. Hawkins et al. (24) observed a higher injury rate among defenders (35%) than midfielders, contrasting with our findings of 30.0% for defenders and 43.6% for midfielders. Arnason et al. (25) found forwards accounted for 22% of injuries in Icelandic and Swedish elite football, exceeding our result of 17.9%.

### 4.2 Injury location by playing position

The significant association between playing position and injured body part (Chi-square = 19.303, *p* = 0.004) reveals distinct injury patterns. Goalkeepers predominantly sustained upper limb injuries (44.4%), consistent with Hawkins et al. (24) and Junge and Dvorak (4), who attributed this to their diving and catching roles. Defenders (94.6%) and midfielders (95.1%) were more prone to lower limb injuries, reflecting the demands of high-intensity, continuous play, as noted by Ekstrand et al. (3) and Waldén et al. (23). Della Villa et al. (13) corroborated these observations, emphasizing that the intensity of high-intensity running and physical demands contribute significantly to injury risks for midfielders. Conversely, Fuller et al. (20) reported a more even distribution of upper and lower limb injuries across positions, suggesting that training and match variability may influence these patterns, warranting further investigation.

### 4.3 Injury type by playing position

Significant differences in injury types (*X*^2^ = 27.692, *p* = 0.001) reveal that soft tissue injuries are most frequent among midfielders (78.0%) and defenders (67.6%), reflecting the high-intensity demands of these roles. This aligns with Arnason et al. (25) and Woods et al. (26), who linked such injuries to repetitive movements and physical strain. Bone-related injuries were more common in goalkeepers (66.7%) and forwards (47.4%) due to sporadic high-impact actions like collisions and falls, as noted by Junge et al. (27) and Hawkins and Fuller (18). However, Hawkins and Fuller (18) suggest that bone injuries in goalkeepers are not significantly higher than in other positions, indicating potential reporting biases or league-specific differences, emphasizing the complexity of injury patterns.

### 4.4 Injury mechanism by playing position

The mechanisms of injury varied significantly across positions, reflecting the diverse demands of the game. Defensive actions, such as tackling, accounted for the majority of injuries among defenders (56.8%), a finding that aligns with studies by Arnason et al. (25) and Hawkins et al. (24), who emphasized tackling as a common and hazardous mechanism in soccer.

Aerial play, including actions such as heading the ball and jumping to contest for possession, was a notable cause of injuries, particularly among goalkeepers. Physical actions, such as collisions, landing from jumps, and running at high intensity, also significantly contributed to the injury mechanisms observed, echoing findings by Bailey et al. (28), who highlighted the unique risks faced by goalkeepers due to their specialized roles. Aiello et al. (29) expanded on these patterns, noting the specific mechanisms leading to injuries in high-intensity actions like saves and aerial duels. Midfielders and forwards exhibited injuries linked to ball control and movement, which were less frequent but nonetheless significant.

The elevated injury risk among midfielders reflects their unique positional demands. High cognitive load, coupled with repetitive high-intensity running and directional changes, significantly contributes to soft tissue overuse injuries, consistent with findings by Della Villa et al. (13) and Woods et al. (26). Goalkeepers' susceptibility to upper limb injuries stems from their reliance on reaction-based actions and aerial challenges, as highlighted by Junge et al. (27) and Bailey et al. (28). These results emphasize how biomechanical and cognitive demands interact to influence injury patterns across positions. Theoretical perspectives suggest that midfielders' roles, involving constant decision-making under physical exertion, further compound their risk of injury (15).

These patterns resonate with earlier research by Rahnama et al. (14) and Twizere (19), who emphasized that the dynamic nature of soccer positions influences the type and mechanism of injuries sustained. Furthermore, a recent studies has highlighted the role of workload management and cumulative physical stress in injury risk, particularly for defenders and midfielders (10, 30). Players with a high acute-to-chronic workload ratio or extensive injury histories are especially vulnerable, underscoring the impact of the physical demands and rapid transitions inherent to these roles. Strategic workload management, including planned rest periods, has been shown to mitigate such risks while maintaining performance (30).

### 4.5 Injury severity by playing position

The severity of injuries did not show statistically significant differences across positions; however, defenders reported a slightly higher proportion of severe injuries (40.5%). This finding is consistent with Junge and Dvorak (4), who noted that high-intensity physical contact in defensive roles increases the likelihood of severe injuries. Recent research supports these observations, highlighting that defenders are more prone to ligament tears due to intense workloads and critical defensive actions (30, 31). Contrastingly, midfielders, despite their high injury frequency, predominantly reported soft tissue injuries, which are generally less severe (24, 31). The economic and performance impact of severe injuries is also well-documented in European soccer, with Pulici et al. (32) estimating significant financial costs associated with ligament and muscle injuries. This underscores the broader implications of injury severity on teams and organizations.

## 5 Practical implication for injury prevention

The findings reinforce the importance of developing position-specific injury prevention strategies. For midfielders, workload management strategies, including recovery protocols and neuromuscular training, are crucial in mitigating overuse injuries. Recent studies have emphasized the role of acute-to-chronic workload ratios in predicting injury risks, highlighting the importance of monitoring player workloads to prevent overexertion (31, 33). For defenders, training programs should prioritize agility, safe tackling techniques, and workload balancing to minimize high-risk situations (30, 31). Goalkeepers, with their unique demands, would benefit from upper limb strengthening, protective gear, and jump technique optimization to address their specific injury risks (30).

Position-specific injury prevention strategies are justified by the differing injury profiles identified in this study. While certain foundational strategies (e.g., agility training and safe tackling) may benefit multiple positions, tailoring interventions to positional demands, such as upper limb strengthening for goalkeepers, ensures a comprehensive approach to injury prevention (10).

## 6 Study limitations and recommendations

The reliance on self-reported data in this study introduces potential recall bias, a limitation commonly noted in retrospective injury epidemiology research (22). To mitigate such biases, recent advancements advocate the use of objective tracking technologies, such as mobile health devices, which provide continuous and accurate data collection, reducing the reliance on subjective recall (34). Moreover, the study's focus on male sub-elite players limits its generalizability to female and elite athletes. Future research could incorporate longitudinal designs, advanced tracking technologies, and a broader demographic scope, as highlighted by recent work emphasizing multilevel and spatially integrated longitudinal models for more comprehensive insights (35).

While this study draws on literature predominantly from elite and professional soccer, its findings contribute novel insights into the underrepresented sub-elite population. Future studies should focus on expanding sub-elite-specific data to strengthen generalizability.

The absence of standardized clinical tools, such as the Oslo Sports Trauma Research Centre questionnaire, is a limitation of the study. This was due to logistical constraints in a multi-institutional setting. Future research should prioritize the use of clinical tools to standardize injury diagnoses and improve data precision.

The sample size for specific playing positions, particularly goalkeepers (9% of the total sample), was limited due to the typical distribution of players in soccer teams. This uneven representation may reduce statistical power and the generalizability of findings for underrepresented positions. Future studies should explore strategies to improve positional representation, such as oversampling goalkeepers or utilizing pooled datasets from multiple studies, to increase statistical power for subgroup analyses.

## 7 Conclusion

The relationship between playing position and soccer injury characteristics is multi-dimensional, with each position presenting distinct injury risks and mechanisms. The findings contribute valuable insights into injury patterns, which can inform the development of targeted prevention strategies aimed at improving player safety and performance over time. The identification of positional factors associated with injury characteristics, align with the Translating Research into Injury Prevention Practice (TRIPP) framework (36). This framework emphasizes the importance of understanding injury mechanisms and risk factors as foundational steps to developing and implementing effective injury prevention strategies tailored to specific contexts.

## Data Availability

The raw data supporting the conclusions of this article will be made available by the authors, without undue reservation.
